# Dereplication of Natural Products with Antimicrobial and Anticancer Activity from Brazilian Cyanobacteria

**DOI:** 10.3390/toxins12010012

**Published:** 2019-12-24

**Authors:** Tania Keiko Shishido, Rafael Vicentini Popin, Jouni Jokela, Matti Wahlsten, Marli Fatima Fiore, David P. Fewer, Lars Herfindal, Kaarina Sivonen

**Affiliations:** 1 Department of Microbiology, University of Helsinki, Viikinkaari 9, FI-00014 Helsinki, Finland; tania.shishido@helsinki.fi (T.K.S.); rafael.popin@helsinki.fi (R.V.P.); jouni.jokela@helsinki.fi (J.J.); matti.wahlsten@helsinki.fi (M.W.); david.fewer@helsinki.fi (D.P.F.); 2 Institute of Biotechnology, University of Helsinki, Viikinkaari 5D, FI-00014 Helsinki, Finland; 3 Center for Nuclear Energy in Agriculture, University of São Paulo, Avenida Centenário 303, 13400-970 Piracicaba, São Paulo, Brazil; fiore@cena.usp.br; 4 Centre for Pharmacy, Department of Clinical Science, University of Bergen, P.O. Box 7804, 5020 Bergen, Norway; lars.herfindal@uib.no

**Keywords:** natural products, mass spectrometry, leukemia, antifungal, antibacterial, cyanotoxins, *Nostoc*

## Abstract

Cyanobacteria are photosynthetic organisms that produce a large diversity of natural products with interesting bioactivities for biotechnological and pharmaceutical applications. Cyanobacterial extracts exhibit toxicity towards other microorganisms and cancer cells and, therefore, represent a source of potentially novel natural products for drug discovery. We tested 62 cyanobacterial strains isolated from various Brazilian biomes for antileukemic and antimicrobial activities. Extracts from 39 strains induced selective apoptosis in acute myeloid leukemia (AML) cancer cell lines. Five of these extracts also exhibited antifungal and antibacterial activities. Chemical and dereplication analyses revealed the production of nine known natural products. Natural products possibly responsible for the observed bioactivities and five unknown, chemically related chlorinated compounds present only in Brazilian cyanobacteria were illustrated in a molecular network. Our results provide new information on the vast biosynthetic potential of cyanobacteria isolated from Brazilian environments.

## 1. Introduction

Cyanobacteria are photosynthetic bacteria commonly found in diverse aquatic and terrestrial environments [1]. They produce a large range of secondary metabolites (natural products) that are predominantly produced to gain evolutionary advantages, such as adaptation to the surrounding environment or as a defense mechanism, rather than being part of primary metabolism (i.e., growth, development, or reproduction) [2,3,4]. Many cyanobacterial natural products are synthesized by non-ribosomal peptide synthetases (NRPS), polyketide synthase (PKS), or hybrid NRPS-PKS (NRPS/PKS) [5]. These molecules have diverse applications in pharmacology, biotechnology, and bioenergy production [6,7,8,9].

Cyanobacterial natural products have a diverse array of biological activities and properties [10,11,12]. Toxins and protease inhibitors are amongst the most commonly reported [13,14,15,16]. Antibiotic activity is frequently found in cyanobacterial extracts, but the active compounds have seldom been determined [17,18,19]. Cyanobacteria are also a promising source of anticancer compounds [20,21]. These include the elastase inhibitor lyngbyastatin [22], the microtubulin inhibitor curacin A [23], the sodium-channel blocker kalkitoxin [24,25], and the antitumor cryptophycin [26]. Although the anticancer activity of cyanobacterial compounds has not been thoroughly investigated, some studies point to diverse implications in apoptosis [27].

Several cyanobacteria produce antileukemic compounds [21,22,23,24,25,26,28]. Approximately 80% of the cases of acute leukemia in adults in the United States are acute myeloid leukemia (AML). Over 29,000 cases of AML were recorded in Europe between 1995 and 2002 [29]. The treatment for this disease is currently based on chemotherapy, and long-term prognosis for such patients is poor [30]. The discovery and development of new drugs to treat AML are necessary due to emerging AML drug resistance and the harsh side effects of the current treatments [31]. Marine cyanobacteria are a rich source of anticancer compounds, but cyanobacteria isolated from underexplored environments are promising alternative source of drug leads for drug discovery [9,27,32,33]. 

Brazilian biomes are characterized by unusually high biodiversity, and new cyanobacterial taxa are frequently described from these biomes [34,35,36,37,38,39]. Strains isolated from these biomes have antimicrobial and anticancer activities [40,41,42]. New natural products are continuously described from cyanobacteria isolated from these habitats [40,43,44]. Metabolic profiling has increasingly been used to visualize and organize the vast chemical diversity and large repertoire of molecules synthesized by microorganisms [45]. This approach enables the assessment of groups of compounds with analogous biosynthetic origins and structures, which may facilitate the discovery of novel compounds [46].

In this study, we screened cyanobacterial isolates from Brazilian habitats for natural products and tested the antileukemic and antimicrobial activity of their culture extracts. Furthermore, we constructed a molecular network to expand the current knowledge of their chemical diversity. 

## 2. Results

### 2.1. Cyanobacterial Isolates

**Figure 1:**
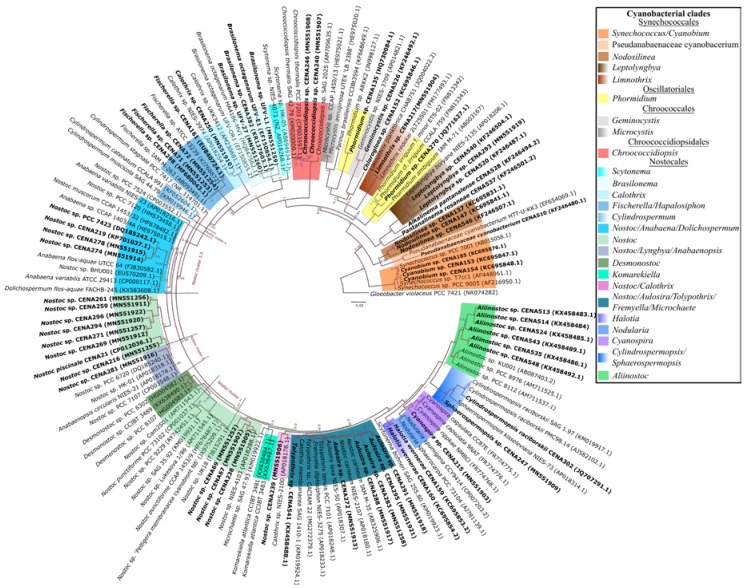
Evolutionary history based on 16S rRNA gene sequences of the 62 analyzed cyanobacterial strains (in bold) and 73 other strains: According to this Bayesian inference, the sequences formed diverse clades belonging to different orders. Accession numbers in NCBI are shown in parentheses.

A total of 62 isolates from diverse biomes from Brazil were tested for antimicrobial and anticancer activities (Table S1). A phylogenetic tree based on 16S rRNA gene was constructed in order to access the evolutionary history of these strains (Figure 1). The 62 isolates belong to 23 different genera spread among five different orders: Synechococcales, Oscillatoriales, Chroococcales, Chroococcidiopsidales, and Nostocales.

### 2.2. Bioactivity Screening

Aqueous and organic cyanobacterial cell extracts were first tested for their cytotoxic activity against the human leukemia MOLM-13 cell line (Table S2). Two of the 62 aqueous extracts resulted in 70–90% cell death (CCIBt3594 and CENA185), while 11 were highly cytotoxic with 90–100% cell death (CENA69, 72, 161, 219, 270, 513, 514, 524, 535, 543, and 548). The organic extracts from the cyanobacteria were more potent, 13 samples induced 70–90% cell death (CENA21, 67, 137, 147, 152, 153, 154, 159, 160, 215, 216, 272, and 526), and 26 induced apoptosis in 90–100% of the cells (CCIBt3594; UFV-E1; UFV-L1; UFV-27; and CENA69, 71, 72, 135, 161, 185, 217, 219, 270, 283, 296, 298, 302, 382, 510, 513, 514, 524, 535, 541, 543, and 548). A dilution of the organic extracts to 3.99 mgDW·ml^-1^ (0.3%) revealed that two samples induced cell apoptosis in 70–90% of the cells (CENA69 and 382) and 13 samples induced apoptosis in 90–100% (CENA71, 72, 161, 185, 219, 270, 296, 298, 513, 514, 524, 535, and 548). The extracts that induced apoptosis between 70–90% of the MOLM-13 cells were diluted 10 times, and the extracts inducing over 90% cell death were diluted 10 and 100 times to further compare the cytotoxic potential. For the aqueous extracts, only *Nostoc* sp. CENA219 presented a moderate apoptosis rate (51%) at 100 times dilution. The organic extracts of *Aliinostoc* sp. CENA69 and CENA513 and *Fischerella* sp. CENA161 were highly cytotoxic (above 70%) towards MOLM-13 cells even at a concentration of 0.133 mgDW mL^−1^ (100-fold dilution).

The cyanobacterial extracts with the highest apoptosis induction of MOLM-13 cells were also tested for cytotoxicity towards the normal rat kidney epithelial NRK cell line (Table 1). By comparing the potency of normal and malignant cells, the test provided evidence for potential use as an anticancer compound. The strains *Fischerella* sp. CENA72, *Cyanobium* sp. CENA185, *Limnothrix* sp. CENA217, *Nostoc* sp. CENA296, and *Aliinostoc* sp. CENA524 showed particularly selective cytotoxicity towards MOLM-13 cells over NRK cells; these strains had an over nine-fold greater EC_50_ value for NRK than MOLM-13 (Table 1). It is noteworthy that the organic extracts from *Cyanobium* sp. CENA185 and *Limnothrix* sp. CENA217 showed no apparent toxicity to NRK cells after 24 h (Figure S1). *Nostoc* sp. CENA69, *Fischerella* sp. CENA161, and *Aliinostoc* spp. CENA513 and CENA514 showed relatively smaller differences in cytotoxicity between the two cell lines as judged by EC_50_ values than the other strains.

**Figure 2:**
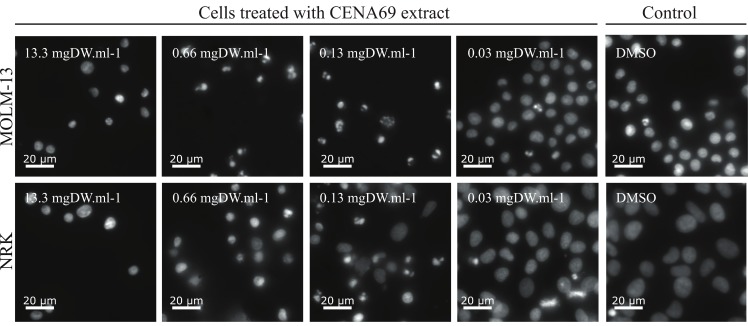
Cytotoxic cell extract from *Nostoc* sp. CENA69: Human leukemia MOLM-13 and rat kidney epithelial NRK cells in different concentrations of cyanobacterial organic extract (indicated as mg of dry weight (DW) of freeze-dried cell mass used per ml of cell suspension). DMSO: dimethyl sulfoxide (negative control). Note the appearance of pyknotic and hypercondenced chromatin in the cells treated with high concentration of CENA69 extract.

Another method to evaluate drug selectivity for one cell over another is to calculate the area under the curve (AUC) of the dose-response curves (Table 1 and Figure S1). *Aliinostoc* sp. CENA514 has a large AUC ratio between NRK and MOLM-13 cell lines, which indicates that the concentrations needed to kill MOLM-13 AML cells will not harm nonmalignant NRK cells. The cell extract from *Nostoc* sp. CENA69 was toxic for both MOLM-13 and NRK cell lines (Figure 2 and Table 1). The low value of the half-maximum effective concentration (EC_50_) of *Nostoc* sp. CENA69 shows the high potency of this cell extract for both tested cell lines (Table 1).

To identify possible antibacterial and antifungal activity, organic extracts obtained from cyanobacterial freeze-dried cells were also tested against *Staphylococcus* *aureus* and *Candida albicans* (Table 2, Files S1 and S2). *Fischerella* spp. CENA71, CENA72, CENA161, and CENA298 and *Aliinostoc* sp. CENA513 showed both antibacterial and antifungal activity. *Aliinostoc* spp. CENA514, CENA535, and CENA548 extracts had antifungal activity while CENA524 presented antibacterial activity.

### 2.3. Natural Products Produced by Brazilian Cyanobacteria

**Figure 3:**
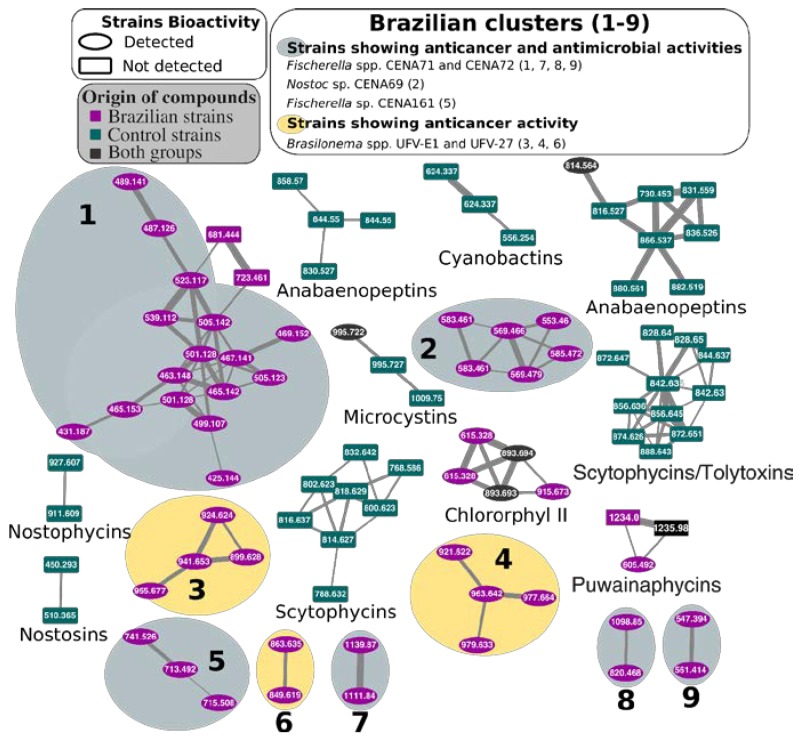
Molecular network of natural products in cyanobacterial extracts from Brazilian (purple) and control (green) strains: Clusters of molecules found only in the former strains are highlighted (1–9). Compound mass/charge ratios (*m/z*) are indicated in the node labels. The complete network can be found in Figure S2.

Cell extracts were analyzed using mass spectrometry, and a molecular network was constructed based on the MS/MS spectra of the detected compounds. Due to the size of the resulting network, only clusters containing potential new novel compounds detected exclusively in Brazilian cyanobacterial extracts and previously known natural products are presented in Figure 3. The complete network can be found in the Supplementary Materials (Figure S2). Cyanobacterial cell extracts from strains producing known compounds were included in the analysis to facilitate the dereplication of different compounds (Table S3). Puwainaphycins produced by *Aliinostoc* spp. CENA535 and CENA548 were detected using a dereplication tool (GNPS) (Figure S3; Table S4).

**Figure 4:**
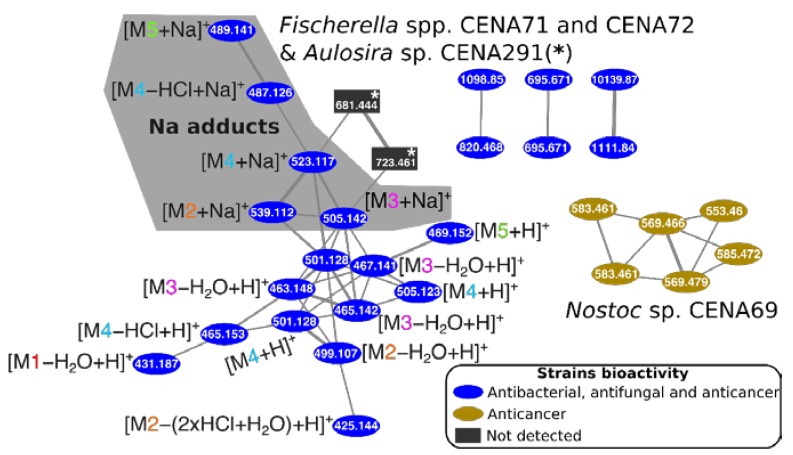
Cluster of compounds in cyanobacterial extracts from Brazilian strains showing potent anticancer or cytotoxic activities (except *Aulosira* sp. CENA291): Compound mass/charge ratios (*m/z*; more than one isotope ion/compound could be present) are indicated in the node labels. See also Figure S4 and File S3.

Cyanobacterial extracts that showed potent anticancer or cytotoxic activity presented novel compounds detected only in Brazilian strains (Figure 4). The chlorinated compounds (M1–5) could not be assigned in this study to any known molecule. Further information on these compounds are presented in the Supplementary Material (Figure S4, File S3).

## 3. Discussion

There has been a recent increase in the number of studies on natural products synthesized by Brazilian cyanobacteria, particularly on toxins and protease inhibitors [43,44,51,52,53]. However, the diversity of these organisms and their natural products will likely expand as these ecosystems are further explored [54,55,56]. Therefore, strains were isolated from different biomes and their biosynthetic potential in culture conditions is discussed here.

The 16S rRNA gene phylogenetic analysis demonstrated that, while some *Nostoc* strains were distributed in the previously described *Nostoc* clusters 1, 2, and 3.3 [43], the remaining strains were located in sparse and diverse clades more closely related to other Nostocalean genera.

Previous research indicated that analysis of a broad selection of taxonomical genera of cyanobacteria would increase the possibility of detecting novel bioactive compounds [21,43]. The analysis of several strains investigated in this study revealed significant antileukemic activity (≥70%). Approximately 21% of the aqueous and 61% of the organic extracts were bioactive, in which all the cyanobacterial strains that produced hydrophilic active compounds also contained a hydrophobic antileukemic extract. The analysis measured by AUC aids in evaluating the effect of the compounds based on concentration and is a reliable method to analyze dose-response curves [57].

Cell extracts of *Cyanobium* sp. CENA154, *Nostoc* spp. CENA67 and CENA69, and *Oxynema* sp. CENA135 have previously been described as having anticancer activity against the murine colon cancer cell line CT-26 and lung carcinoma 3LL [42]. In the present work, cell extracts of these strains were similarly found to induce apoptosis of the AML cell line MOLM-13. Their organic extract is responsible for the antileukemic activity, except for *Nostoc* sp. CENA69, in which the aqueous extract also exhibited antileukemic activity at 13.3 mgDW mL^-1^ concentration. CENA69 showed a high potency to induce cell death in MOLM-13 and was further tested using normal cells (NRK), which also showed high cytotoxicity. *Fischerella* spp. CENA71 and CENA72 and *Aliinostoc* spp. CENA513, CENA514, and CENA524 produce antileukemic compound(s) with higher potency against MOLM-13 cells than normal NRK cells. A similar study that investigated extracts from Portuguese cyanobacteria also revealed effects against cancer cells but did not reduce the viability of NRK cells [58].

Among the 39 strains exhibiting high antileukemic activity, 12 of them produce previously described compounds. *Cyanobium* spp. CENA153 and CENA185, *Fischerella* sp. CENA161, *Oxynema* sp. CENA135, and *Phormidium* sp. CENA270 produce microcystins. These peptides are potent hepatotoxins due to their protein serine/threonine phosphatase inhibitor activity [59]. Hepatocytes efficiently take up microcystin and are therefore the primary target of toxicity [60]. However, high doses of microcystin can also induce oxidative stress [61]. The cellular effects of protein phosphatase inhibitors, such as microcystin and nodularin, are due to differences in uptake. These toxins do not distinguish between malignant and nonmalignant cells if microinjected into the cells [62]. Nevertheless, previous studies have shown that there is no correlation between phosphatase inhibitors and apoptogenic activity against SH-SY5Y-neuroblastoma and AML cells [58,63]. The lipopeptides hassallidin and puwainaphycin have been previously shown to have cytotoxic activity [64,65]. Aeruginosin and anabaenopeptin are protease inhibitors with no reported cytotoxic effects. Lastly, saxitoxin is a neurotoxin that is associated with cytotoxic effects on mammalian cells [66,67,68]. Further analysis using isolated compounds would be necessary to show which are the antileukemic compounds produced by the tested Brazilian cyanobacteria. Three of the 62 strains are likely to produce new antibacterial, antifungal, or anticancer molecules.

The construction of a molecular network was used to explore the complex variety of natural products synthesized by the analyzed cyanobacteria. Although most of the molecules are produced by both Brazilian and control strains, approximately 20% of the resulting clusters are exclusively present in the group of Brazilian strains. Compounds that had already been identified in the strains, such as microcystin-LR (*Fischerella* sp. CENA161) [48], [D-Leu^1^]microcystin-LR (*Phormidium* sp. CENA270) [49], anabaenopeptin, and nodularin (*Aliinostoc* sp. CENA543) [43], were again found here (Figure S2 and Table S4). The molecular network also highlighted molecules that are possibly related to their bioactivities, though the mass spectrometry analysis was not sufficient for identification and further isolation and characterization are necessary.

Previous studies involving 17 of the 62 strains tested here (CENA21, 67, 69, 135, 152, 153, 154, 159, 160, 185, 216, 217, 247, 271, 281, 283, and 510) reported anticancer and antimicrobial activity on other cell lines and microorganisms [41,42,47]. Thus, our analysis expands the number of strains producing known bioactive compounds, especially from the *Nostoc* and *Fischerella* genera [32,43,52]. *Nostoc* is a cosmopolitan genus of cyanobacteria that is predominantly terrestrial but is also frequently found in symbiosis with other organisms [69,70]. They are known as a prolific producer of secondary metabolites, such as peptides, polyketides, and alkaloids [71,72]. The compounds already described include microcystin (hepatotoxin) [73], nostophycin (cytotoxic activity) [74], cryptophycin (tumor inhibitor) [75], nostocyclamide (algae and bacteria growth inhibitor) [76], nostocarboline (acetylcholinesterase inhibitor) [77], nostocine A (algae and plant growth inhibitor) [78], muscoride A (antibacterial activity) [79], and cyanobacterin (cyanobacterial growth inhibitor) [80]. Nevertheless, the biosynthetic potential of *Nostoc* remains underestimated, as many compounds have not yet been identified [65]. Although *Fischerella* is comparatively less studied that *Nostoc*, several natural products have been identified, such as microcystin [43], the antitumor welwitindolinones [81], the antibacterial and antimycotic hapalindoles [82], and the antibacterial ambiguine [83].

## 4. Conclusions

Brazilian cyanobacteria belonging to 5 different orders and isolated from different biomes showed anticancer, antibacterial, and antifungal activity. The known compounds microcystin, saxitoxin, aeruginosin, hassallidin, nodularin, anabaenopeptin, pseudoaeruginosin, and puwainaphycin were detected by mass spectrometry analysis and a dereplication tool. Nonetheless, the biosynthetic potential of the strains remains mostly unknown as most of the molecular network could not be assigned to known natural products. Among these compounds, some were found only in Brazilian strains and could potentially be involved in the detected bioactivities. The unknown chlorinated molecules detected in extracts showing relatively high anticancer activity are promising candidates for isolation and characterization. We hypothesize that further studies with Brazilian cyanobacteria may reveal an even larger biosynthetic repertoire, as the tested strains are expected to represent just a small fraction of isolation sources in the ecosystems in the country.

## 5. Materials and Methods

### 5.1. Cyanobacterial Strains

*Nostoc* spp. CENA67 and CENA69 and *Fischerella* spp. CENA71 and CENA72 (No. 3–6 in Table S1) were isolated from the anthropogenic Amazon Dark Earth soils, which are highly fertile anthropogenic soils produced by native populations of Central Amazonia [84]. *Phormidium*, *Nodosilinea*, *Chlorogloea*, *Cyanobium*, and *Halotia* strains (No. 7–14 in Table S1) originate from Cardoso Island on the coast of the State of São Paulo. This region is covered by the Atlantic Forest, mangroves, and restingas (herbaceous vegetation) and is part of one of the most biodiverse ecosystems in the world [85]. Caatinga, the biome in which *Aulosira, Calothrix, Chroococcidiopsis, Cyanospira*, *Fischerella, Lyngbya*, *Nostoc*, and *Phormidium* strains (No. 17–43 in Table S1) were isolated, is a semiarid region with singular adapted species that is largely neglected by conservation policies in Brazil [54]. Lastly, *Alkalinema*, *Geminocystis*, *Leptolyngbya*, *Nodosilinea*, *Nostoc*, *Pannus*, and *Pantanalinema* strains (No. 1 and 46–59 in Table S1) were isolated from the Pantanal, which is the largest continental wetland on the planet and is colonized by unique cyanobacteria [54]. The strains are maintained in the UHCC culture collection of the University of Helsinki, Helsinki, Finland, and the culture collection of CENA/University of São Paulo, Piracicaba, São Paulo, Brazil.

### 5.2. DNA Extraction and Sequencing, and Phylogenetic Analyses

Total genomic DNA of the strains was extracted from 3-weeks old cultures according to the method by Fiore et al. [86]. The 16S rRNA genes were obtained by PCR using the set primer 27F-23S30R [87]. PCR products were cloned and sequenced, and the resulting reads were assembled into contigs using the Phred/Phrap/Consed package [88,89,90].

The evolution model GTR + I + G was selected as the best-fit model by jModelTest 2 v0.1.1 [91,92] and was therefore used for inferring the 16S rRNA phylogenetic tree. In total, 141 cyanobacterial nucleotide sequences were implemented in MrBayes v3.2.6 [93] with 5,000,000 generations.

### 5.3. Cyanobacterial Extracts

The Brazilian strains selected for the bioactivity assays (Table S1) were cultivated in 3 L of Z8 [94] medium (with or without 0.467 g/L of NaNO_3_ as nitrogen source; see Table S5) under constant light of 10 ± 1 µmol m^−2^ s^−1^ at 20 ± 1 °C. Cells in exponential phase of growth (21–28 days of cultivation) were freeze-dried, and 100 mg was used for methanol extraction. To perform the extraction, 1 mL of methanol and glass beads (0.5-mm diameter, Scientific Industries INC, Bohemia, NY, USA) were added to the cell matter to be disrupted in a plastic tube using a FastPrep™-24 homogenizer (Fastprep^®^-24, MpBiomedicals, Irvine, CA, USA) for three times of 30 s at 6.5 m/s. The sample tubes were centrifuged at 10,000× *g* for 5 min, and the supernatant was reserved in a new tube. The extraction was repeated. The supernatant from both extractions was combined and dried using a vacuum concentrator (RVC 2–18 CDplus, Martin Christ, Osterode, Germany). Samples were resuspended in 500 μL of methanol (Sigma-Aldrich, San Luis, MO, USA) by vortexing and pipetting. Dichloromethane (Merck) and milli-Q water were added 1:1 *v*/*v* (500 μL:500 μL), and the samples were inverted to mix the solvents. The samples were then centrifuged at 10,000× *g* for 5 min at 20 °C. The aqueous and organic phases were collected, placed in different tubes, and left with the lid open at room temperature in a laboratory fume hood overnight. The aqueous phase was evaporated in a speed-vac (Eppendorf) at 30 °C. Dried samples were stored at −20 °C. Organic-phase extracts were resuspended in 75 μL of dimethyl sulfoxide (DMSO, Sigma-Aldrich, ≥99.9%, A.C.S. spectrophotometric grade), while the aqueous phase was resuspended in 300 μL of 25% DMSO (25 DMSO:75 milli-Q water). Samples were stored at −20 °C.

Frieze-dried cells (100 mg) were extracted twice with 1 mL of methanol and centrifuged as described above. The extracts (300 µL) were added to discs and evaporated overnight. The discs were used for antibacterial and antifungal assays. A volume of 40 µL of filtered (22 µm) extract was mixed with 160 µL of methanol to be analyzed by mass spectrometry and stored at −20 °C. Frieze-dried cells (10 to 24.3 mg) from 10 cyanobacteria (Table S3) were extracted once with 1 mL of methanol as described above, and 200 µL of the extract was filtered and reserved for mass spectrometry analysis.

### 5.4. Cell-Based Assay

The acute myeloid leukemia (AML) cell line MOLM-13 (ACC, 554; [95]) and rat kidney epithelial cell line NRK (ATCC, CRL-6509) were used. Aqueous (4 μL) and organic (1 μL) cyanobacterial extracts were cultured in RPMI or DMEM medium, respectively, and both were supplemented with fetal bovine serum (FBS, F7524, Sigma-Aldrich) and penicillin and streptomycin (P0781, Sigma-Aldrich). The MOLM-13 cells were maintained at concentrations between 10^5^ and 8 × 10^5^ cells/mL. NRK cells were detached by mild trypsin treatment at 70–90% confluence and reseeded at 30–50% confluence. The cells were incubated in a humified atmosphere at 37 °C and 5% CO_2_. To test for cytotoxicity, extracts were added in 96-well plates (Nunclon^TM^ Delta Surface, Thermo Scientific) and MOLM-13 cell suspensions (25–40 × 10^5^ cells/mL) were added to a final volume of 0.1 mL in each well. NRK cells were seeded in wells the day before the experiment (7–8 × 10^4^ cells/mL) and left overnight to attach. Fresh medium containing the cyanobacterial cell extracts were added to the wells. Both cell lines were incubated at 37 °C for 24 h before the cells were fixed by adding 100 μL of 3.7% formaldehyde in phosphate-buffered saline buffer, pH 7.4. The DNA-specific dye Hoechst 33342 (0.01 mg/mL) was then added. Plates were kept at 4 °C overnight, and nuclear morphology was assessed as previously described [96] using a Zeiss Axio Vert.a1 microscope. In brief, we scrutinized Hoechst-stained nuclei and scored them as normal or abnormal. Normal nuclei were moderately stained and bean-shaped, whereas nuclei from dead (apoptotic or necrotic) cells were typically hypercondensed, fragmented, and not bean-shaped. See Figure 2 and Reference [96] for examples and details. The numbers of normal and abnormal nuclei were used to determine the percentage of cell death. At least 100 cells were counted to determine cytotoxicity.

The extracts were first screened for cytotoxic potential, and based on the cytotoxic potential of those inducing more than 50% cell death (50–70%, 70–90%, and above 90%), dilutions series were made to estimate EC_50_ values. All extracts were tested in duplicates. EC_50_ values were calculated, and dose-response curves were created using the nonlinear regression function of GraphPad Prism 8 (GraphPad Software, La Jolla, CA, USA). The program was also employed for calculating *p*-value using the extra-sum-of-squares F test.

### 5.5. Antifungal and Antibacterial Assays

The activity of crude extracts obtained from Brazilian cyanobacteria were tested for antifungal (*Candida albicans* HAMBI484) and antibacterial (*Staphylococcus aureus* HAMBI66) activities. The antifungal test was performed as previously described [32]. PDA and BHI media were used for the growth and activity assay of *Candida albicans* HAMBI484 (at 35 °C) and *Staphylococcus aureus* HAMBI66 (at 37 °C), respectively. Microbial cells grown overnight on solid media were added to sterile water in a sterile tube, and turbidity was measured at 530 nm to approximately McFarland 0.5. The *S. aureus* HAMBI66 inoculum (800 µL) was spread onto BHI plates and dried for 30 min in sterile conditions. *C. albicans* HAMBI484 inoculum was spread using a sterile swab. The discs containing dry cyanobacterial cell extracts were added to the plates and incubated at 35 °C or 37 °C overnight, after which the inhibitions zones were measured. Nystatin (25 µg) and kanamycin (1000 µg, Abtek biological) were used as positive controls against *C. albicans* HAMBI484 and *S. aureus* HAMBI66, respectively.

### 5.6. Liquid Chromatography-Mass Spectrometry (LC-MS)

Samples were injected into an Acquity UPLC system (Waters, Manchester, UK) equipped with a Kinetex^®^ C8 100 LC Column (50 × 2.1 mm, 1.7 µm, 100 Å). The UPLC was operated with a flow-rate of 0.3 mL/min in gradient mode at a temperature of 40 °C. Solvents used in the gradient were A: 0.1% formic acid in water and B: 0.1% formic acid in acetonitrile/isopropanol (1:1). The initial conditions of the linear gradient were A: 95% and B: 5% and the conditions were changed to A: 0% and B: 100% in 5 min. The injection volume was 0.5 μL. Mass spectra were recorded with a Waters Synapt G2-Si mass spectrometer (Waters, Manchester, UK). Measurements were performed using positive and negative electrospray ionization (ESI) in resolution mode. Precursor ions were scanned in the range from *m/z* 400 to 2000 and productions from *m/z* 50 to 2000. MS analyses were performed with scan times of 0.1 s and MS/MS analyses with scan times of 0.2 s. The capillary voltage was 1.5 kV (2.0 kV in negative ionization), source temperature was 120 °C, sampling cone was 40.0, source offset was 80.0, desolvation temperature was 600 °C, desolvation gas flow was 1000 L/h, and nebulizer gas flow was 6.5 Bar. Leucine-encephalin was used as a lock mass, and calibration was performed with sodium formate and Ultramark 1621.

### 5.7. Molecular Network

The data obtained with LC-MS was converted to mzXML using MSConvert [97]. A molecular network was obtained using the GNPS: Global Natural Products Social Molecular Network website [98]. The data was filtered, peaks with + or −17 Da of the precursor were removed, and peaks throughout the spectrum other than the top six +/−50 Da were filtered out. The data were then clustered with MS-Cluster with a parent mass tolerance of 0.2 Da and a MS/MS fragment ion tolerance of 0.2 Da to create consensus spectra. Consensus spectra that contained less than two spectra were discarded. A network was then created where edges were filtered to have a cosine score above 0.7 and more than six matched peaks. Further edges between two nodes were kept in the network only if each of the nodes appeared in each other’s respective top 10 most similar nodes. The spectra in the network were then searched against the GNPS spectral libraries. The library spectra were filtered in the same manner as the input data. All matches kept between network spectra and library spectra were required to have a score above 0.7 and at least six matched peaks.

The *in silico* peptidic natural product dereplicator tool from the GNPS website was used to search for potential known compounds produced by Brazilian cyanobacteria. The precursor and fragment ion mass tolerance selected was 0.02 Da, and a search for analogs was performed (VarQuest).

### 5.8. Data Availability Statement

The 16S rRNA or 16S-23S rRNA sequence accession numbers in NCBI are *Nostoc* spp. CENA67 (MN551902), CENA69 (MN551252), CENA216 (MN551255), CENA238 (MN551905), CENA239 (MN551906), CENA259 (MN551911), CENA261 (MN551256), CENA269 (MN551912), CENA271 (MN551257), CENA274 (MN551914), CENA278 (MN551915), CENA281 (MN551916), and CENA294 (MN551920); *Leptolyngbya* CENA293 (MN551919); *Fischerella* spp. CENA71 (MN551253) and CENA 72 (MN551254); *Cyanospira* sp. CENA215 (MN551903); *Limnothrix* sp. CENA217 (MN551904); *Chroococcidiopsis* spp. CENA240 (MN551907) and CENA246 (MN551908); *Sphaerospermopsis* sp. CENA247 (MN551909); *Calothrix* sp. CENA250 (MN551910); *Aulosira* spp. CENA272 (MN551913), CENA283 (MN551258), CENA288 (MN551917), CENA291 (MN551918), and CENA295 (MN551921); Nostocales CENA296 (MN551922); and *Brasilonema* spp. UFV-L1 (MN551259) and UFV-27 (MN551260).

## Figures and Tables

**Table 1:** Comparison of inhibition caused by organic cyanobacterial extracts in rat kidney epithelial NRK and human leukemia MOLM-13 cell lines as measured by the area under the curve (AUC) of cells with normal nuclei and EC_50_. See methods Section 5.3 for calculations of EC_50_ and AUC.

Cyanobacterial Strain	AUC					EC_50_	
	NRK		MOLM-13		Ratio		
	Total Area	Std. Error	Total Area	Std. Error		NRK	MOLM-13
*Nostoc* sp. CENA69	63.01	2.902	26.61	4.412	2.4	0.098	0.054
*Fischerella* sp. CENA71 *	106.3	9.467	45.45	4.053	2.3	1.493	0.363
*Fischerella* sp. CENA72 *	164.4	14.78	52.32	9.073	3.1	3.129	0.201
*Fischerella* sp. CENA161	134.0	6.417	69.02	7.495	1.9	0.508	0.083
*Cyanobium* sp. CENA185	199.7	-	110.7	−	−§	>15#	1.590
*Limnothrix* sp. CENA217	198.6	1.033	137.7	3.028	−§	>15#	1.410
*Nostoc* sp. CENA296 *	172.5	15.93	96.91	4.076	1.8	11.26	1.212
*Fischerella* sp. CENA298	124.9	8.210	98.90	12.37	1.3	2.283	1.324
*Aliinostoc* sp. CENA513	129.2	6.390	53.58	5.328	2.4	0.491	0.087
*Aliinostoc* sp. CENA514 *	108.2	4.706	32.96	5.631	3.3	0.842	0.140
*Aliinostoc* sp. CENA524 *	159.6	4.367	65.36	4.124	2.4	6.012	0.620
*Aliinostoc* sp. CENA535	85.71	2.182	60.54	10.19	1.4	1.218	0.566

Range given due to missing data in intermediate toxicity (see Figure S1); * *p*-value < 0.0001 calculated using extra-sum-of-squares F test in GraphPad; # Cells were not affected with highest concentration tested. § Value could not be calculated due to a lack of activity in NRK cells.

**Table 2:** Summary of the antifungal (F), antibacterial (B), and anticancer (C) activities observed in the cyanobacterial crude extracts (see also Files S1 and S2). Bioactive cell extracts (+) showed inhibition zone in the antimicrobial assay and above 70% induction of apoptosis of MOLM-13 cell lines at the concentration of 13.3 mgDW mL^−1^. Previously obtained information of the compounds produced by the studied strains are presented. Cyanobacterial extracts that induced apoptosis in less than 70% of MOLM-13 cells did not present inhibition halo in the antimicrobial assays or that were not previously described to produce a natural product were not included in this table. The use of sterile water may have influenced the susceptibility of fungal and bacterial cells.

Taxon	Strain	Activity			Compounds Detected	Reference
		F	B	C ^a^		
*Pannus brasiliense*	CCIbt3594	−	−	+	−	This study
*Nostoc piscinale*	CENA21	−	−	+	−	This study
*Nostoc* sp.	CENA67	−	−	+ *	−	[42] and this study
*Nostoc* sp.	CENA69	−	−	+ *	−	[42] and this study
*Fischerella* sp.	CENA71	+	+	+	−	This study
*Fischerella* sp.	CENA72	+	+	+	−	This study
*Oxynema* sp.	CENA135	−	−	+ *	Microcystin, Saxitoxin	[41,42] and this study
*Nodosilinea* sp.	CENA137	−	−	+	−	this study
*Nodosilinea* sp.	CENA147	−	−	+	−	this study
*Chlorogloea* sp.	CENA152	−	−	+	−	this study
*Cyanobium* sp.	CENA153	−	−	+	Microcystin, Aeruginosin	[41,47] and this study
*Cyanobium* sp.	CENA154	−	−	+ *	Aeruginosin	[42,47] and this study
*Halotia wernerae*	CENA159	−	−	+	−	This study
*Halotia wernerae*	CENA160	−	−	+	−	This study
*Fischerella* sp.	CENA161	+	+	+	Microcystin	[48] and this study
*Cyanobium* sp.	CENA185	−	−	+	Microcystin	[41] and this study
*Cyanospira* sp.	CENA215	−	−	+	−	This study
*Nostoc* sp.	CENA216	−	−	+	−	This study
*Limnothrix* sp.	CENA217	−	−	+	−	This study
*Nostoc* sp.	CENA219	−	−	+	Hassallidin	[32] and this study
*Phormidium* sp.	CENA270	−	−	+	Microcystin	[49] and this study
*Nostoc* sp.	CENA271	−	−	−	Aeruginosin	[47] and this study
*Gloeotrichia* sp.	CENA272	−	−	+	−	This study
*Calothrix* sp.	CENA283	−	−	+	−	This study
*Nostoc* sp.	CENA296	−	−	+	−	This study
*Fischerella* sp.	CENA298	+	+	+	−	This study
*Cylindrospermopsis raciborskii*	CENA302	−	−	+	Saxitoxin	[50] and this study
*Brasilonema* sp.	CENA382	−	−	+	−	This study
*Pseudanabaenaceae cyanobacterium*	CENA510	−	−	+	−	This study
*Aliinostoc* sp.	CENA513	+	+	+	−	This study
*Aliinostoc* sp.	CENA514	+	−	+	−	This study
*Aliinostoc* sp.	CENA524	−	+	+	−	This study
*Geminocystis* sp.	CENA526	−	−	+	−	This study
*Aliinostoc* sp.	CENA535	+	−	+	Puwainaphycin	This study
*Tolypothrix* sp.	CENA541	−	−	+	−	This study
*Aliinostoc* sp.	CENA543	−	−	+	Nodularin, Anabaenopeptin, Pseudoaeruginosin	[11,43] and this study
*Aliinostoc* sp.	CENA548	+	−	+	Puwainaphycin	This study
*Brasilonema octagenarum*	UFV-E1	−	−	+	−	This study
*Brasilonema* sp.	UFV-L1	−	−	+	−	This study
*Brasilonema* sp.	UFV-27	−	−	+	−	This study

^a^ Cytotoxic towards MOLM-13 cells (see Table 1).*Anticancer activity has also been previously observed [40].

## Supplementary Materials

The following are available online at https://www.mdpi.com/2072-6651/12/1/12/s1, Figure S1: Dose-response curves of the selected cyanobacterial extracts on NRK (Blue) and MOLM-13 (red) cell lines: These were the basis for estimating EC_50_ values, Figure S2: Complete molecular networking of cyanobacterial extracts from Brazilian (purple) and control (green) strains: Known compounds are indicated (see also Tables S3 and S4). Node numbers represent mass/charge ratios (*m/z*), Figure S3: MS/MS spectra of puwainaphycins identified in *Aliinostoc* sp: CENA535 using the dereplication tool (GNPS). See Table S4 for further information, Figure S4: Spectra of potentially new compounds produced only by Brazilian strains (see also Figure 4 and File S3), Table S1: List of Brazilian cyanobacterial isolates tested for the synthesis of anticancer and antimicrobial compounds: The accession number of their 16S rRNA sequences and the habitat, site, city, and state from where the samples were collected are indicated, Table S2: Human leukemia MOLM-13 cells apoptosis caused by crude extracts of aqueous and organic cyanobacterial cell extracts (average of two parallel measurements indicated in %). Dilutions (0.3×, 0.1×, and 0.01×) are indicated in the table, and samples not tested in different dilutions are indicated with (−). Red color indicates high amounts of apoptotic cells, and green indicates high amounts of normal cells. The error (Er) indicates the difference value of the average with one of the duplicates, Table S3: List of cyanobacteria used as a control in the LC-MS analysis to compare the previously discovered compounds with the molecules produced by Brazilian strains, Table S4: Dereplicator analysis of Brazilian cyanobacteria cell extracts. Predicted compounds are organized by *p*-value (cutoff of 10^−11^); FDR cutoff of 15%. Compounds found in previous studies are in bold, File S1: Pictures of the antifungal activity essays using *Candida albicans* HAMBI484: The use of sterile water may have influenced the susceptibility of the fungal cells, File S2: Pictures of the antibacterial activity essays using *Staphylococcus aureus* HAMBI66: The use of sterile water may have influenced in the susceptibility of the bacterial cells, File S3: Retention times of chlorinated compounds detected in *Fischerella* sp. CENA72, Table S5: Culture media used for the cultivation of the strains: The addition of nitrogen refers to 0.467 g/L of NaNO_3_.
