# Supplementary material for: Dereplication of Natural Products with Antimicrobial and Anticancer Activity from Brazilian Cyanobacteria
Source: Toxins (Basel). 2019 Dec 24;12(1):12. doi: 10.3390/toxins12010012 (PMC7020483; doi:10.3390/toxins12010012)
Supplement: Supplementary file 1 [file toxins-12-00012-s001.zip › toxins-672330 supple correct/FileS1 Pictures of the antifungal activity essays using Candida albicans HAMBI484.pdf]

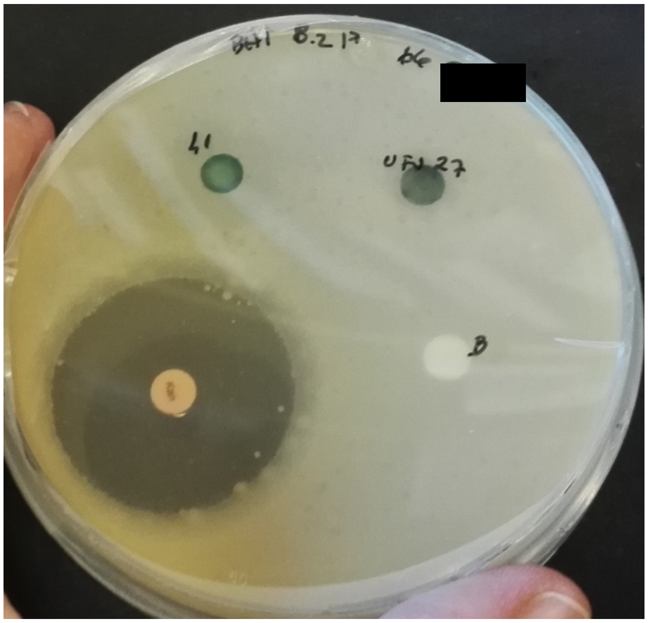

CENA41, UFV-27, Positive control, Negative control

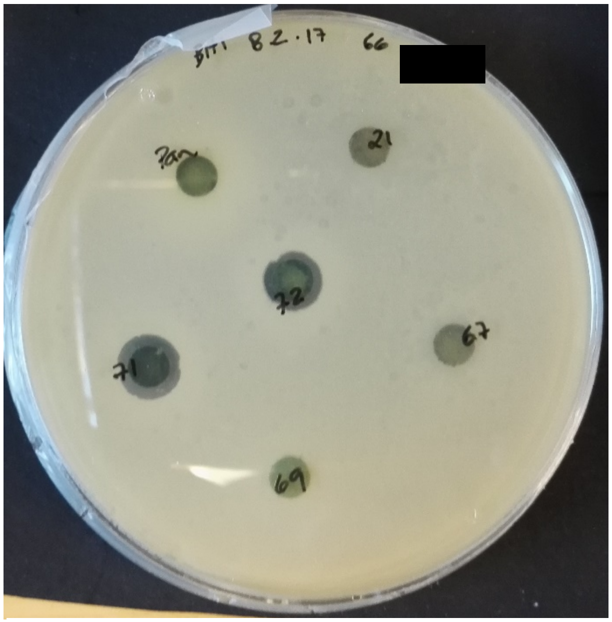

CCIBt3594, CENA21, CENA71, CENA72, CENA67, CENA69

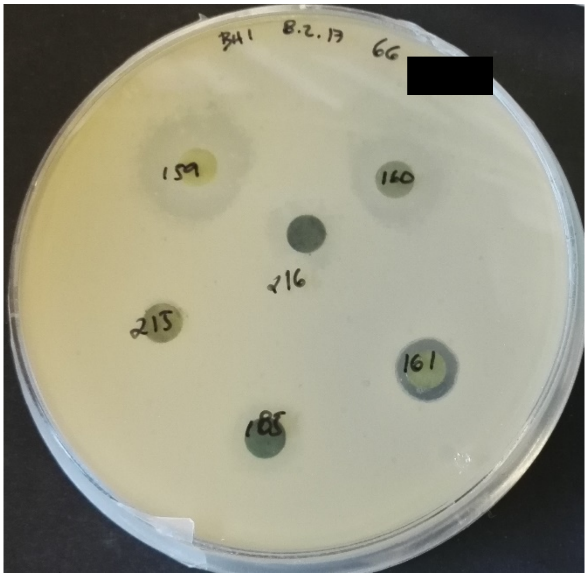

CENA159, CENA160, CENA216, CENA 215, CENA185, CENA161

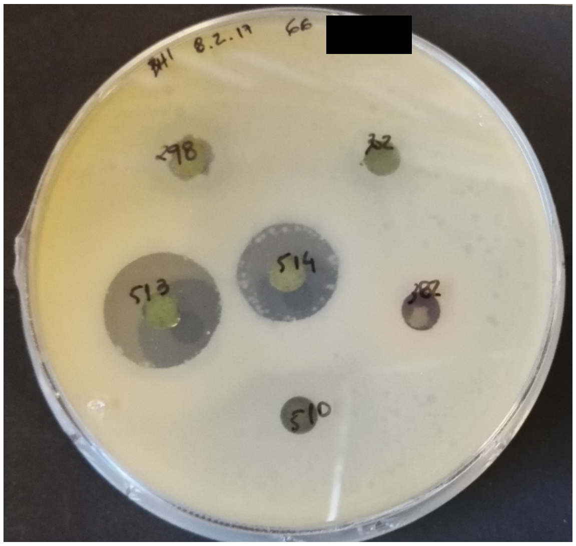

CENA298, CENA302, CENA513, CENA514, CENA382, CENA510

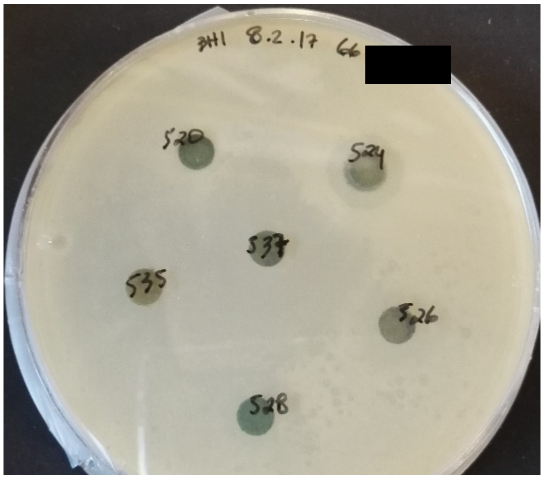

CENA520, CENA524, CENA535, CENA537, CENA526, CENA528

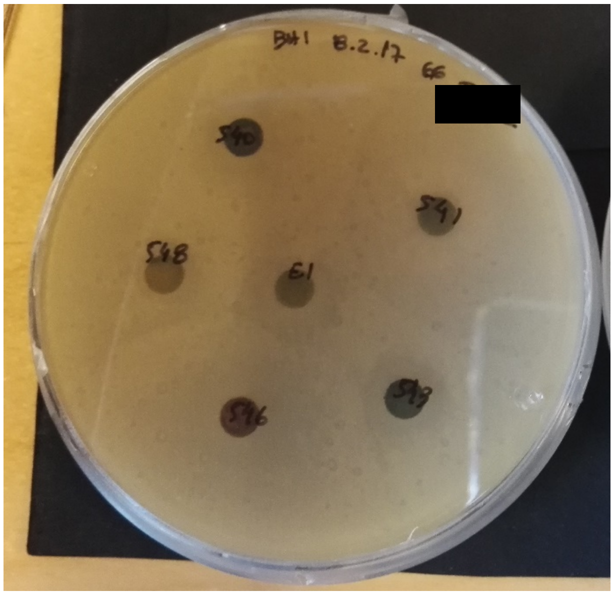

CENA540, CENA541, CENA548, UFV-E1, CENA546 and CENA543.

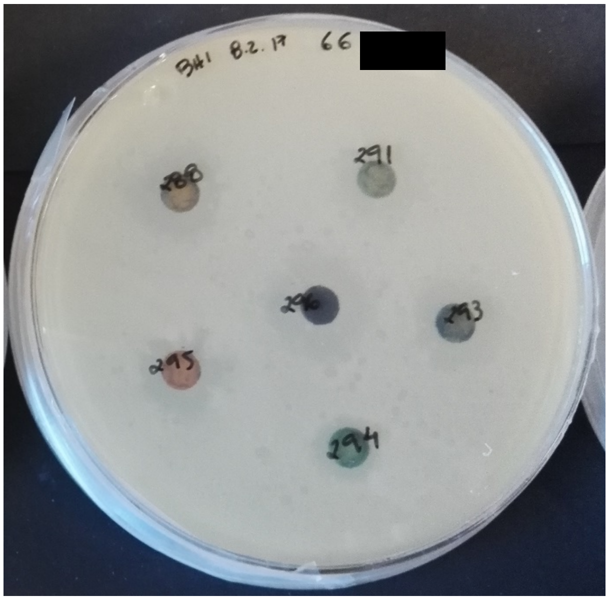

CENA288, CENA291, CENA295, CENA296, CENA293, CENA294

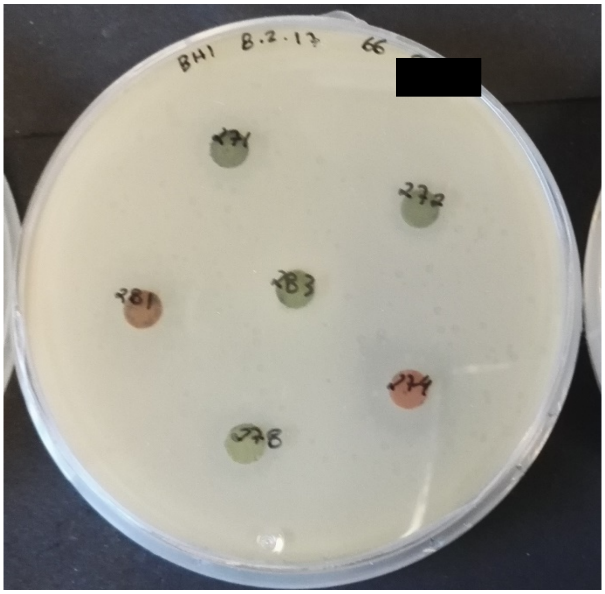

CENA271, CENA272, CENA281, CENA283, CENA278, CENA274

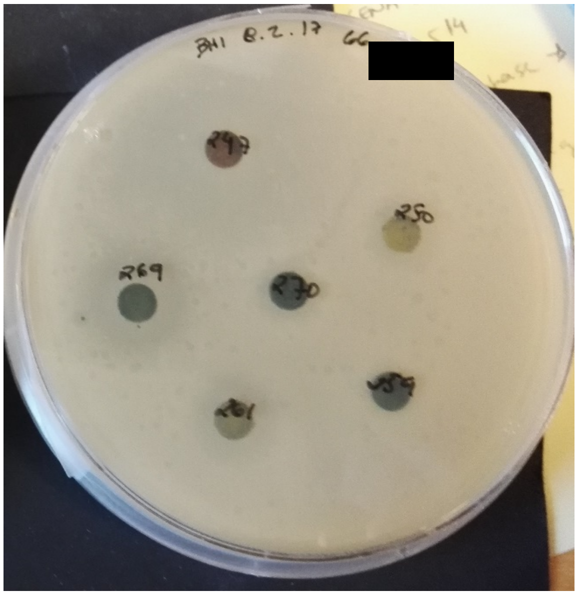

CENA247, CENA250, CENA269, CENA270, CENA261, CENA259

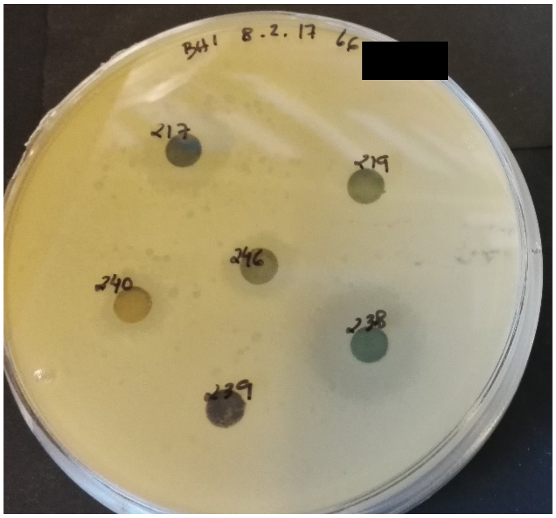

CENA217, CENA219, CENA240, CENA246, CENA239, CENA238

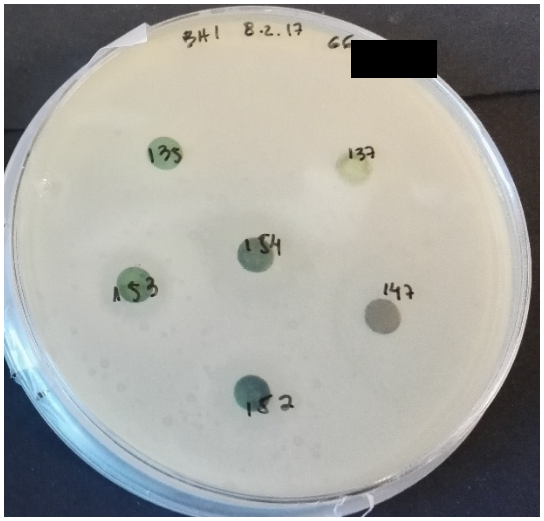

CENA135, CENA137, CENA153, CENA154, CENA152, CENA147
