# Supplementary material for: Dereplication of Natural Products with Antimicrobial and Anticancer Activity from Brazilian Cyanobacteria
Source: Toxins (Basel). 2019 Dec 24;12(1):12. doi: 10.3390/toxins12010012 (PMC7020483; doi:10.3390/toxins12010012)
Supplement: Supplementary file 1 [file toxins-12-00012-s001.zip › toxins-672330 supple correct/FileS2 Pictures of the antibacterial activity essays using Staphylococcus aureus HAMBI66.pdf]

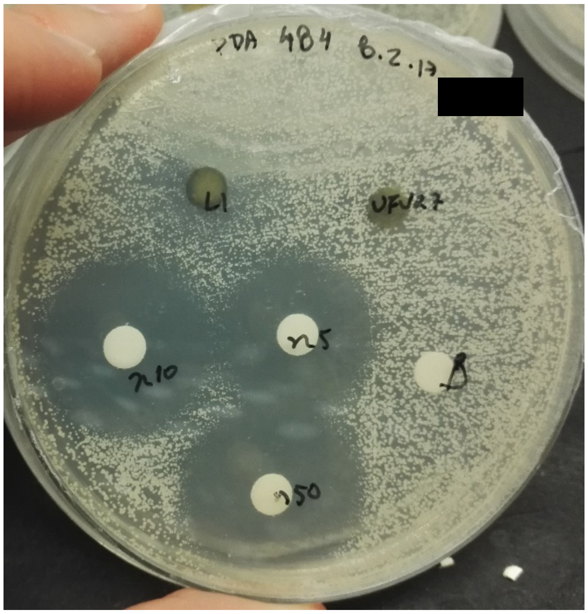

UFV-L1, UFV-27, Positive Controls, Negative Control

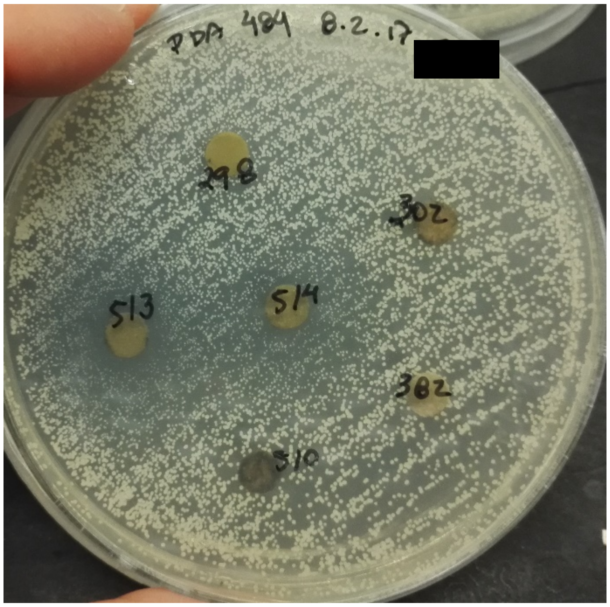

CENA298, CENA302, CENA513, CENA514, CENA382, CENA510

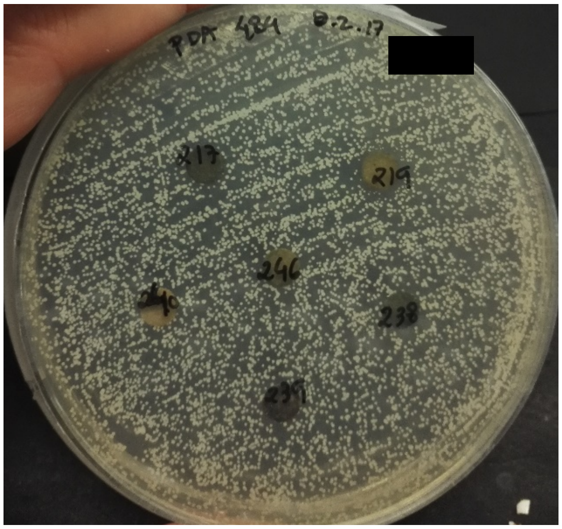

CENA217, CENA219, CENA240, CENA246, CENA238, CENA239

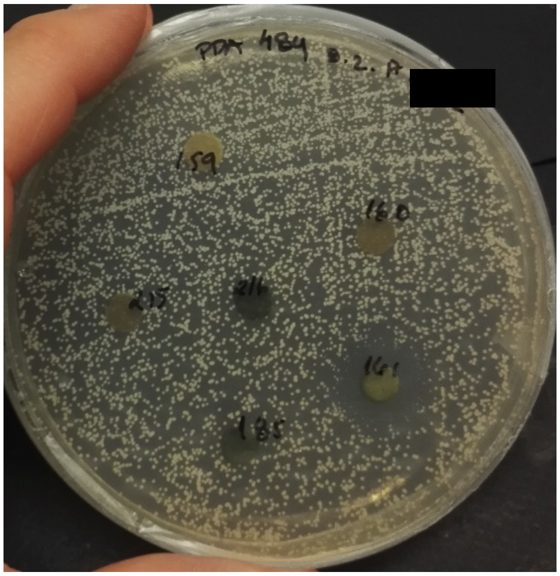

CENA159, CENA160, CENA215, CENA216, CENA161, CENA185

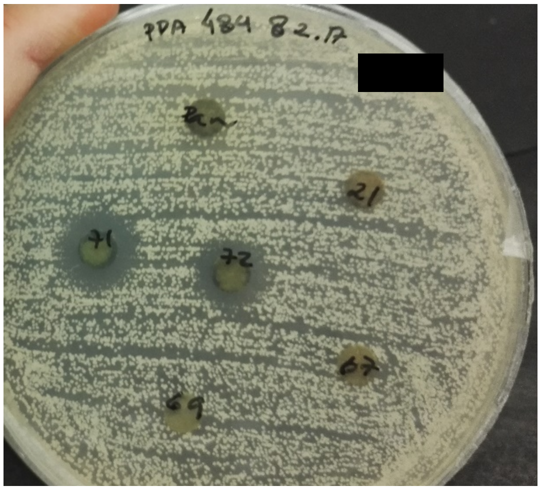

CCIBt3594, CENA21, CENA71, CENA72, CENA67, CENA69

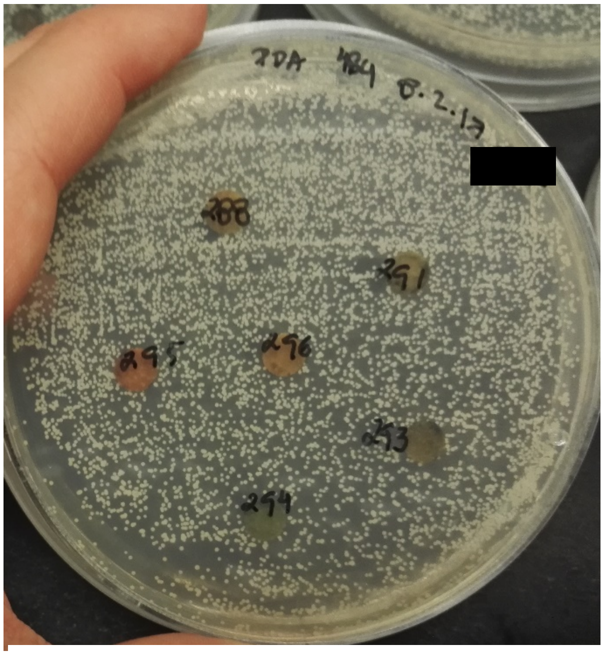

CENA288, CENA291, CENA295, CENA296, CENA293, CENA294

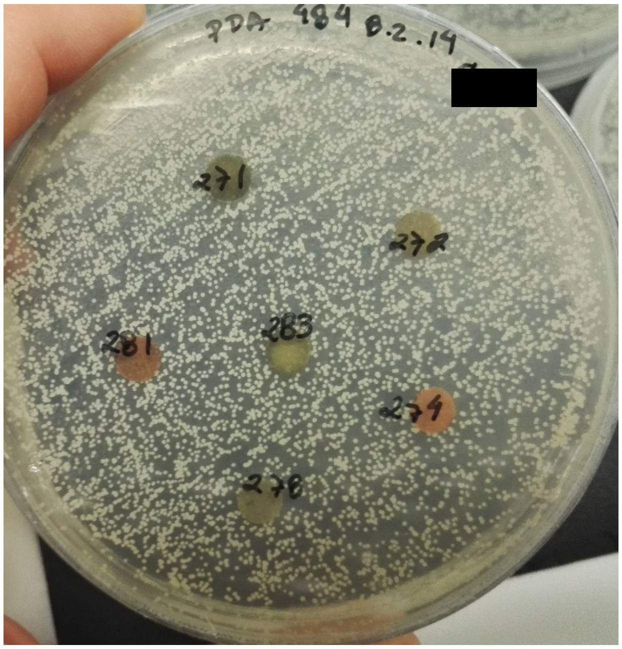

CENA271, CENA272, CENA281, CENA283, CENA274, CENA278

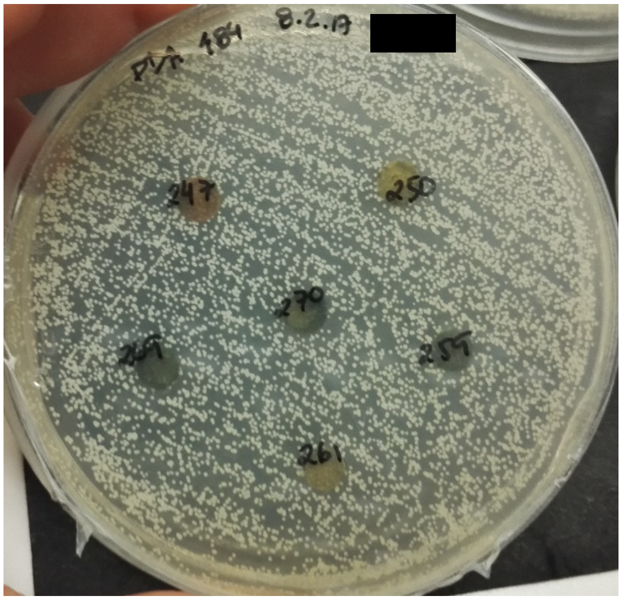

CENA247, CENA250, CENA269, CENA270, CENA259, CENA261

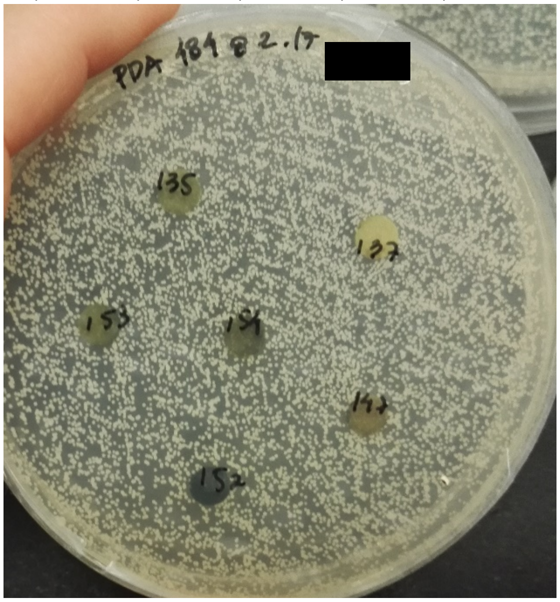

CENA135, CENA137, CENA153, CENA154, CENA147, CENA152

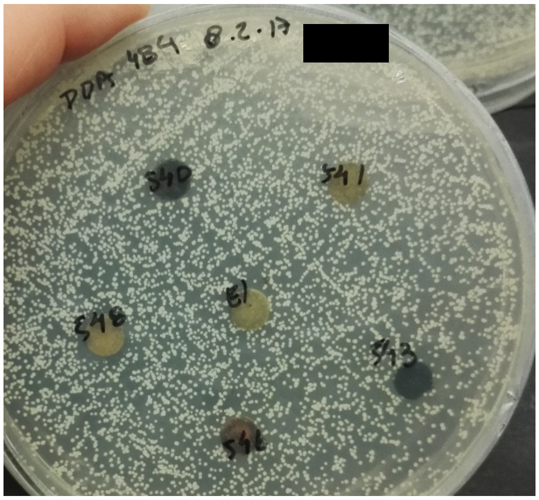

CENA540, CENA541, CENA548, UFV-E1, CENA513, CENA543

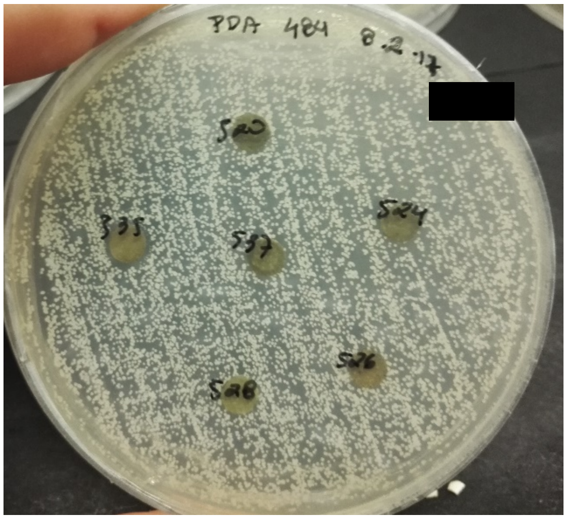

CENA520, CENA535, CENA537, CENA524, CENA528, CENA526
