# Supplementary figures and images for: Dereplication of Natural Products with Antimicrobial and Anticancer Activity from Brazilian Cyanobacteria
Source: Toxins (Basel). 2019 Dec 24;12(1):12. doi: 10.3390/toxins12010012 (PMC7020483; doi:10.3390/toxins12010012)

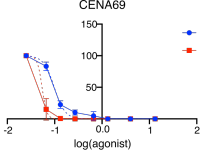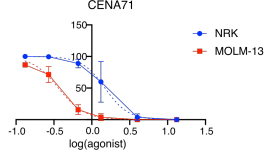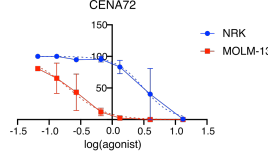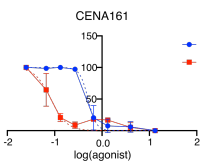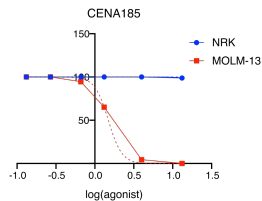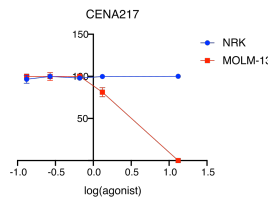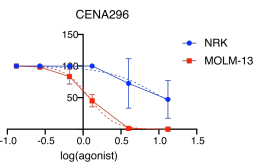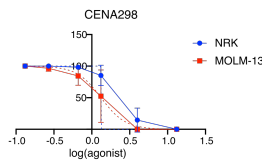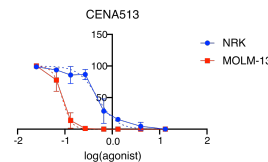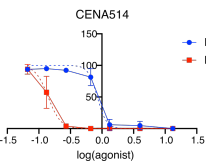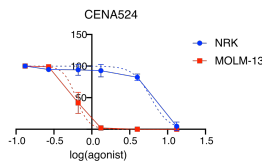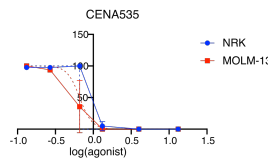

Supplement: Supplementary file 1 [file toxins-12-00012-s001.zip › toxins-672330 supple correct/FigureS1 Dose-response curves of the selected cyanobacterial extracts on NRK (Blue) and MOLM-13 (red) cell lines.pdf]

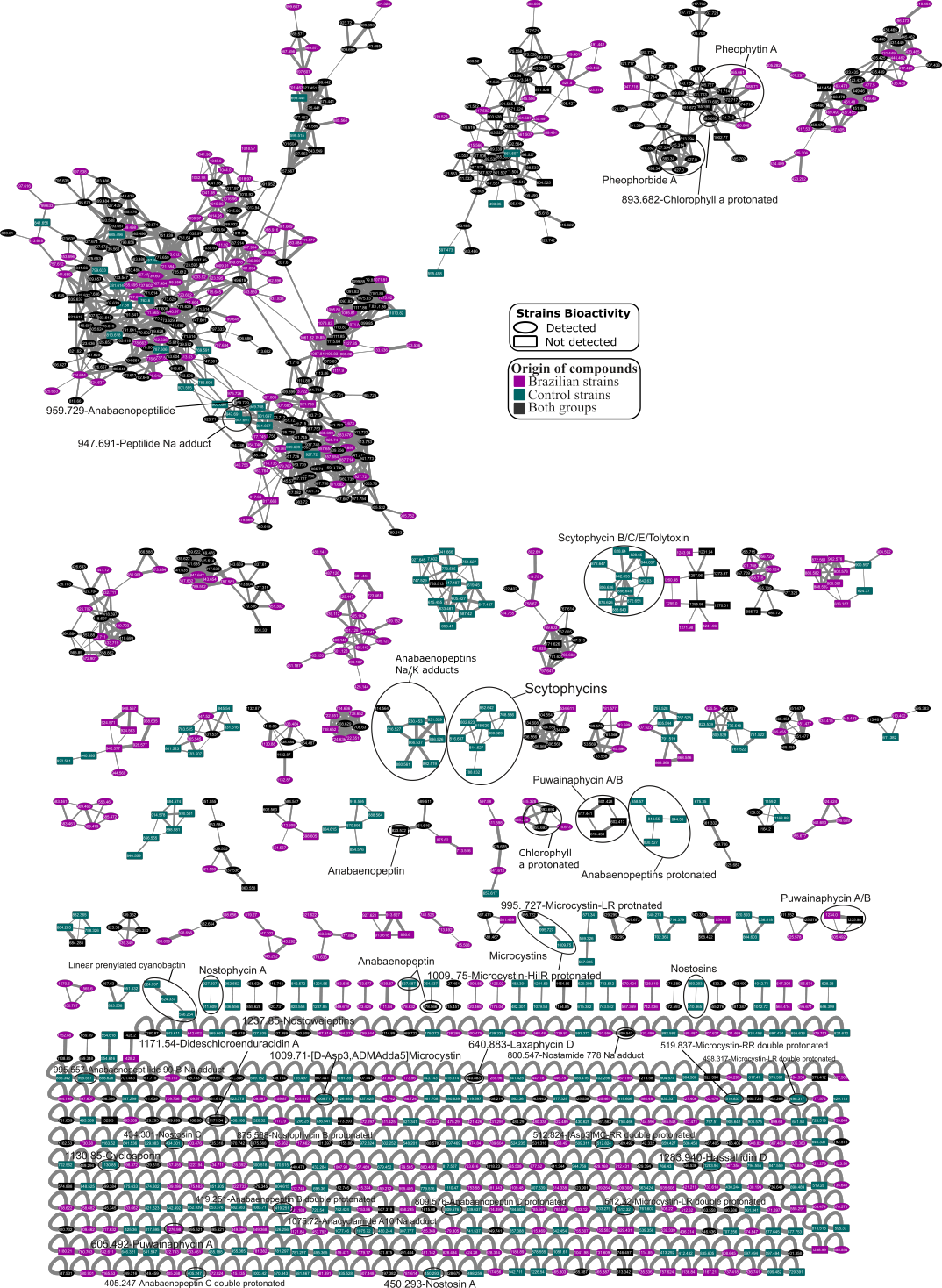

Supplement: Supplementary file 1 [file toxins-12-00012-s001.zip › toxins-672330 supple correct/FigureS2 Complete molecular networking of cyanobacterial extracts from Brazilian (purple) and control (green) strains.pdf]

# *Aliinostoc* sp. CENA535

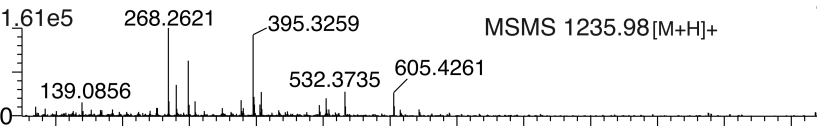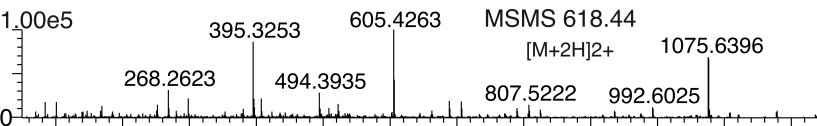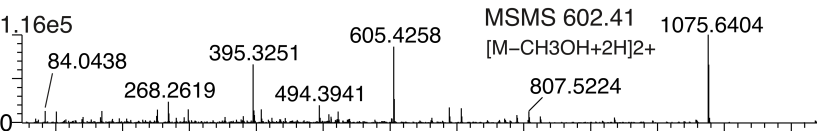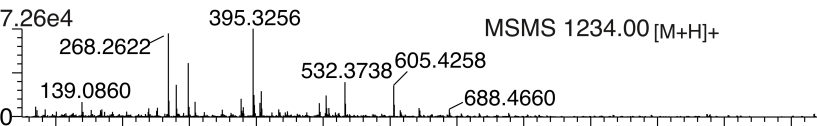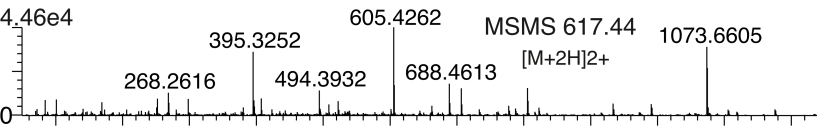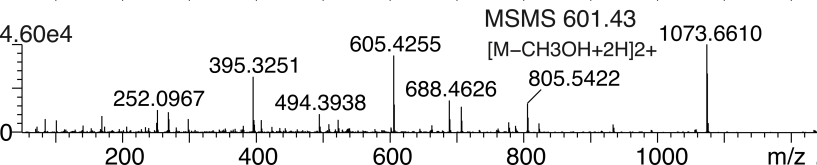

Puwainaphycin A

Puwainaphycin B

Supplement: Supplementary file 1 [file toxins-12-00012-s001.zip › toxins-672330 supple correct/FigureS3 MS/MS spectra of puwainaphycins identified in Aliinostoc sp_ CENA535 using the dereplication tool (GNPS).pdf]

# Fischerella sp. CENA72

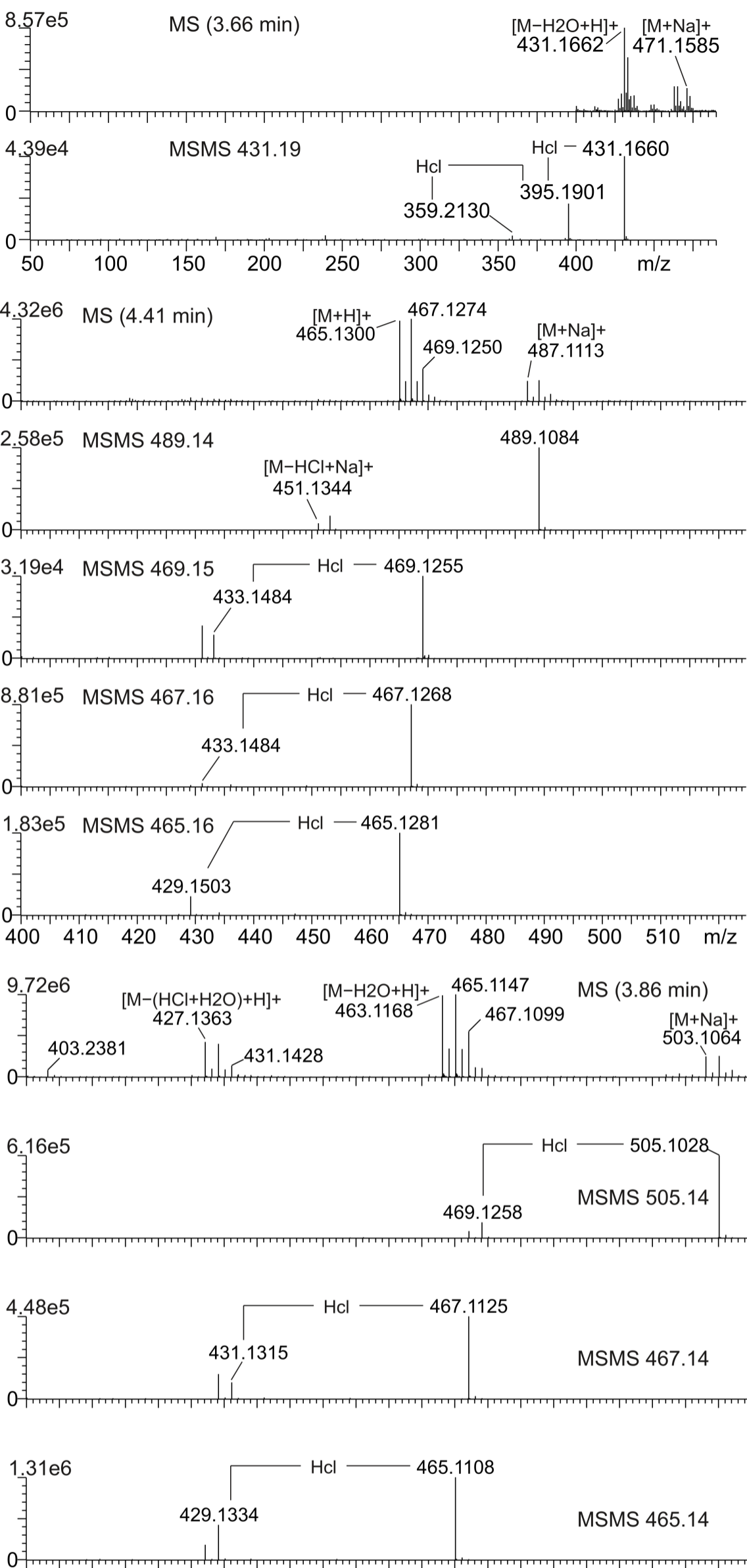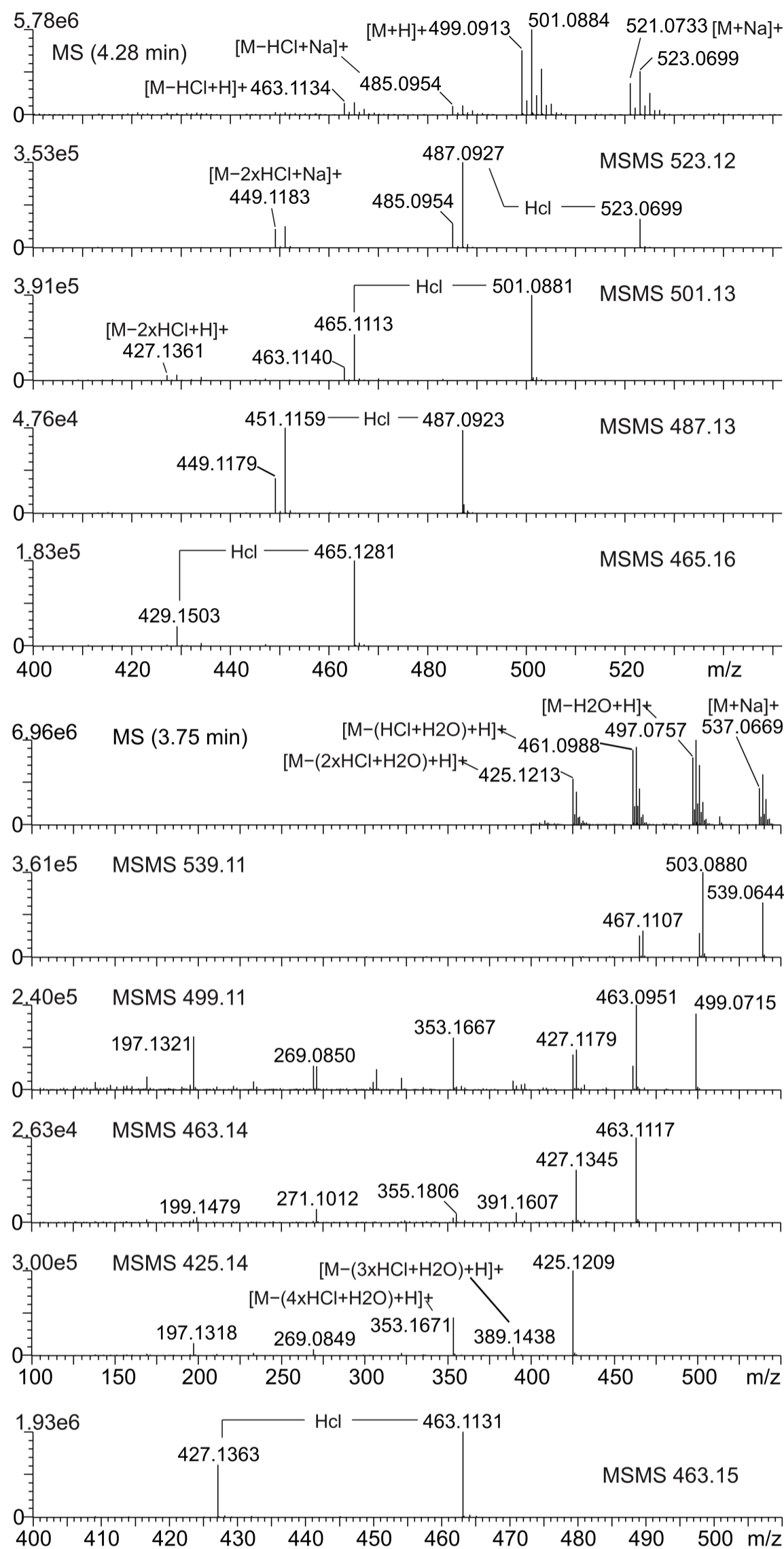

# Nostoc sp. CENA69

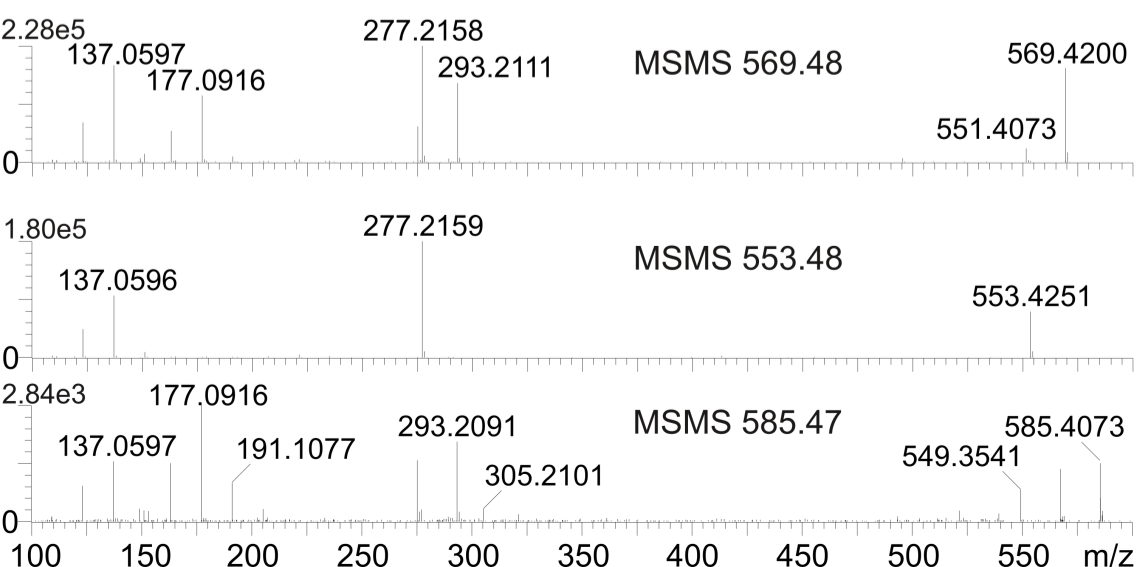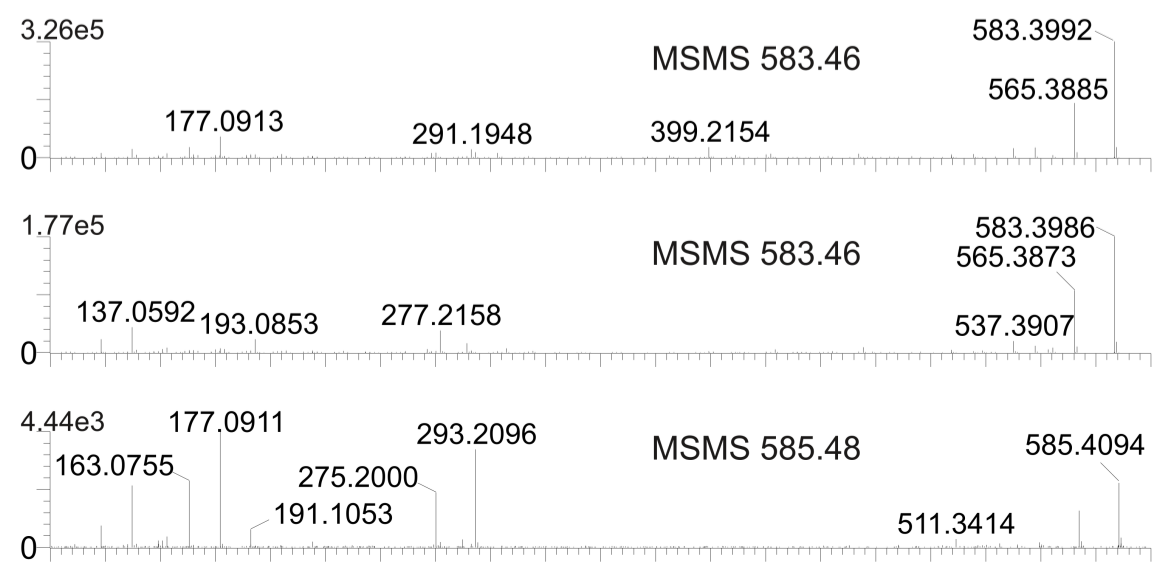

Supplement: Supplementary file 1 [file toxins-12-00012-s001.zip › toxins-672330 supple correct/FigureS4 Spectra of potentially new compounds produced only by Brazilian strains.pdf]
